# Author Correction: Miniaturized and untethered McKibben muscles based on photothermal-induced gas-liquid transformation

**DOI:** 10.1038/s41467-024-47003-2

**Published:** 2024-03-26

**Authors:** Wenfei Ai, Kai Hou, Jiaxin Wu, Yue Long, Kai Song

**Affiliations:** 1 Key Laboratory of Bio-inspired Materials and Interfacial Science, Technical Institute of Physics and Chemistry, CAS, Beijing, 100190 P. R. China; 2 School of Future Technology, University of Chinese Academy of Sciences, Beijing, 100049 P. R. China; 3 Binzhou Institute of Technology, Weiqiao-UCAS Science and Technology Park, Binzhou City, Shandong Province 256606 China

**Keywords:** Chemistry, Soft materials

Correction to: *Nature Communications* 10.1038/s41467-024-45540-4, published online 13 February 2024

In the original version of the published article, the Acknowledgements section the grant number was in the wrong order. The correct Acknowledgement paragraph is:

W.A., K.H., J.W., Y.L., and K.S. were supported by the Ministry of Science and Technology of China (No. 2019YFA0709200 and 2022YFA1503000); the National Natural Science Foundation (21988102); and TIPC Director’s Fund.

This has now been corrected both in the HTML and the PDF versions of the article.

