# Supplementary material for: Miniaturized and untethered McKibben muscles based on photothermal-induced gas-liquid transformation
Source: Nat Commun. 2024 Feb 13;15:1329. doi: 10.1038/s41467-024-45540-4 (PMC10864313; doi:10.1038/s41467-024-45540-4)
Supplement: Supplementary file 1 — Supplementary Information [file 41467_2024_45540_MOESM1_ESM.pdf]

Supplementary Information

Miniaturized and untethered McKibben muscles based on photothermal-induced gas-liquid transformation

Wenfei Ai [1,2,4], Kai Hou[1,4], Jiaxin Wu [1,2], Yue Long[1,3,*], Kai Song[1,2,3,*]

[1] Key Laboratory of Bio-inspired Materials and Interfacial Science, Technical Institute of Physics and Chemistry, CAS, Beijing 100190, P. R. China

[2] School of Future Technology, University of Chinese Academy of Sciences, Beijing 100049, P. R. China

[3] Binzhou Institute of Technology, Weiqiao-UCAS Science and Technology Park, Binzhou City, Shandong Province, 256606, China

[4] These authors contributed equally to this work: Wenfei Ai, Kai Hou.

[*]Corresponding authors. E-mail addresses: longyue@mail.ipc.ac.cn; songkai@mail.ipc.ac.cn

This PDF file includes:
Supplementary Figs. 1 to 10
Other supplementary materials for this manuscript include the following:
Supplementary Movies 1 to 6

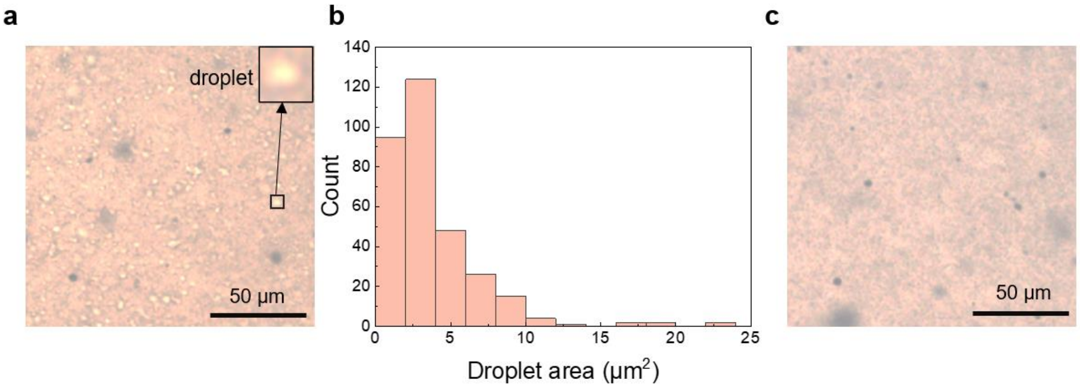

Supplementary Fig. 1. Optical microscopic images of different prepolymers. a Optical microscope image of prepolymerized liquid containing low boiling point liquid droplets. **b** Distribution of droplets area. **c** Optical microscope image of liquid without liquid droplets.

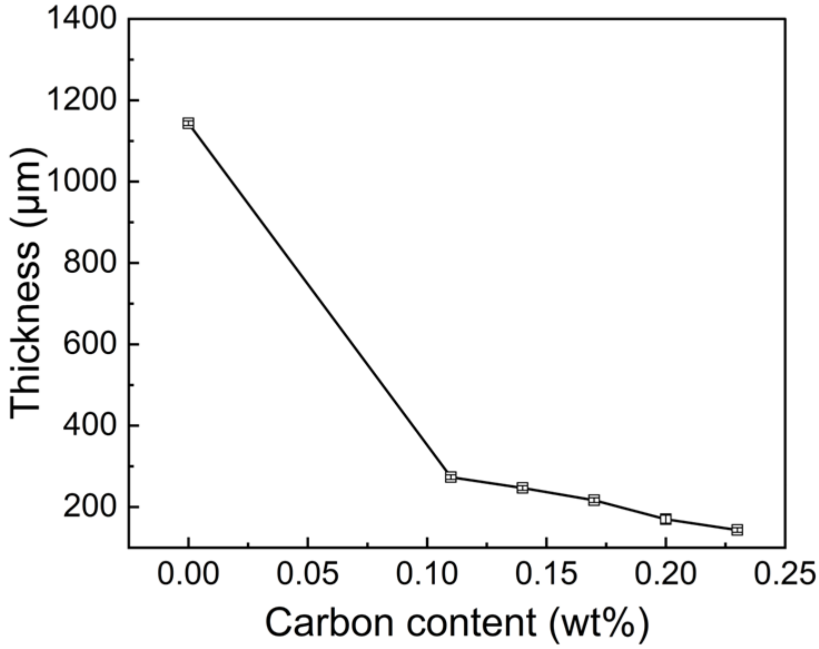

Supplementary Fig. 2. Artificial muscle shell thickness changes with carbon content. With the increase of carbon content, the thickness of artificial muscle shell decreased, and the thickness changed significantly when the carbon content increased from 0 to 0.10 wt%. Even when the thickness of the shell gradually decreasing, the stretchability of the film still gradually increased due to the effect of particle reinforcement. All the error bars correspond to s.d.(n = 3)

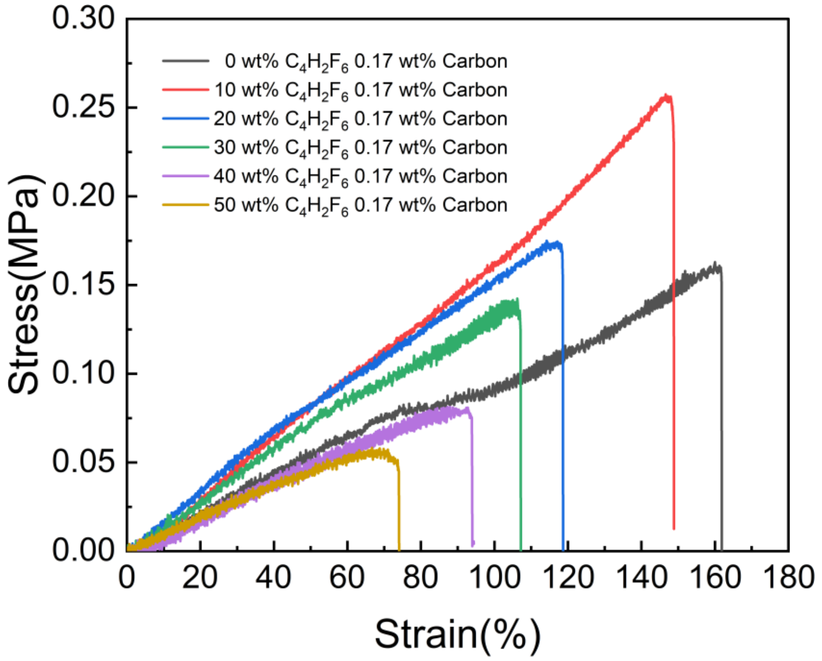

Supplementary Fig. 3. Stress-strain of artificial muscle with 0 - 50 wt% liquid content (increased by 10% intervals), and 0.17 wt% carbon content. The increase in liquid content leads to the decrease of the degree of crosslinking in the artificial muscle shell, and therefore its maximum strain decreases

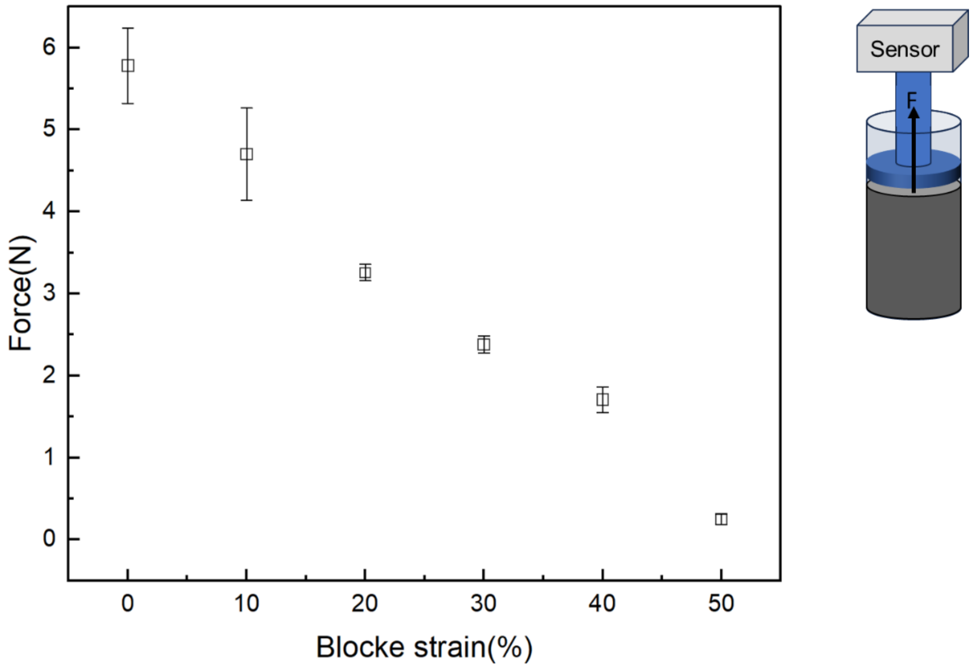

Supplementary Fig. 4. The blocking force of cylindrical artificial muscle at different elongations. All the error bars correspond to s.d. (n = 3)

When the artificial muscle is heated and stretched under light, the direction of force on the connection point is shown in Fig. S5. 2) above, assuming $F_1 = F_2$. When the left foot is pushed, because the direction of F_1 is inclined downward, a vertical downward force $F_{1,\mathrm{a}}$ is generated, and the friction force f_L=u ($F_G + F_{1,\mathrm{a}}$), the direction is shown in Figure 2). Similarly, since the direction of F is inclined upward when the right foot is pushed, the vertical upward force $F_{2,\mathrm{a}}$ is generated, and the friction force $f_R = (F_G - F_{2,\mathrm{a}})$, so $f_R < f_L$, combining with the above conditions, the left foot moves less than the right foot in the light. When the light is turned off, the artificial muscle recovered, and the direction of force on the left and right feet was opposite to that in Fig. S5. 2) $F_R = (F_G + F_{2,\mathrm{a}})$, the retracted distance of the left foot is smaller than that of the right foot. In summary, in a complete light switch turn, the small artificial muscle device walks to the right as a whole. At the same time, in order to increase the initial friction difference between the left and right feet, a zigzag shape is designed on the right foot to increase its friction.

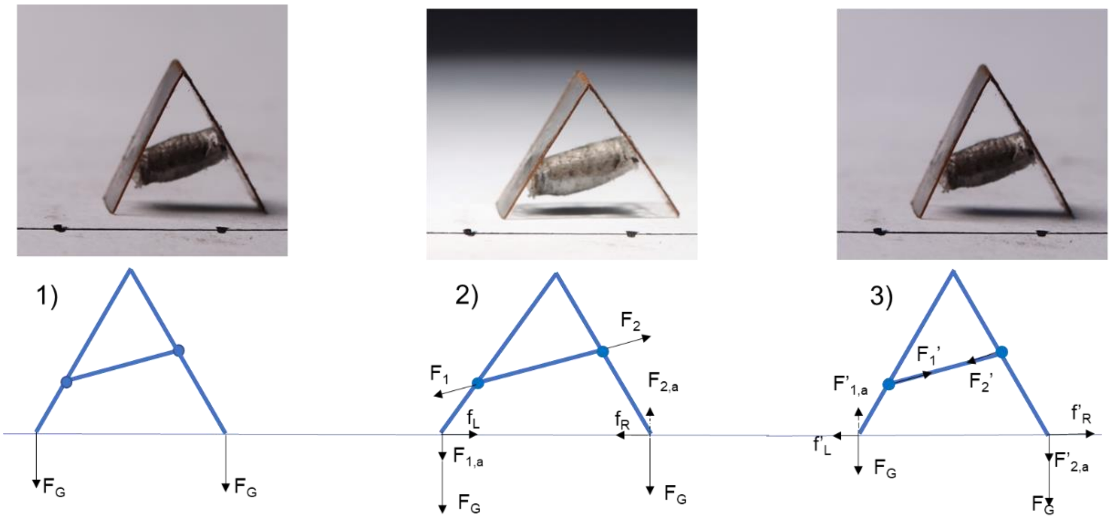

Supplementary Fig. 5. Force analysis of the stress process of walking miniature artificial muscle device.

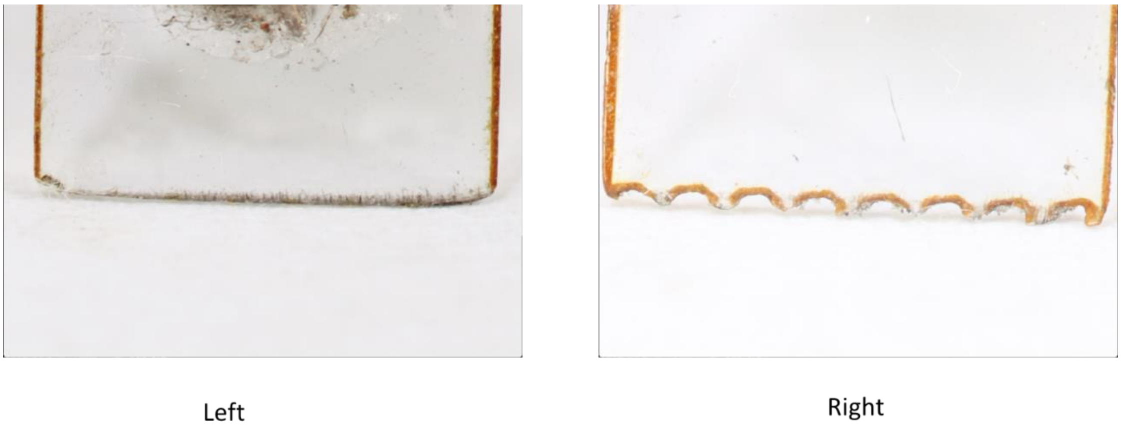

Supplementary Fig. 6. Optical image of the left and right feet of a miniature artificial muscle walking device, respectively.

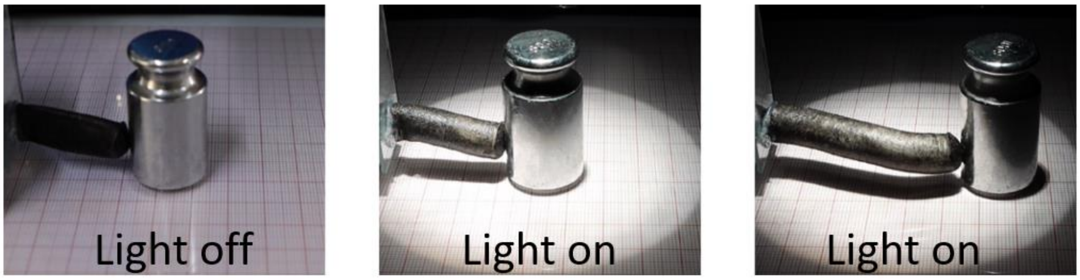

Supplementary Fig. 7. The artificial muscle pushed a 100g weight 2.1cm under light.

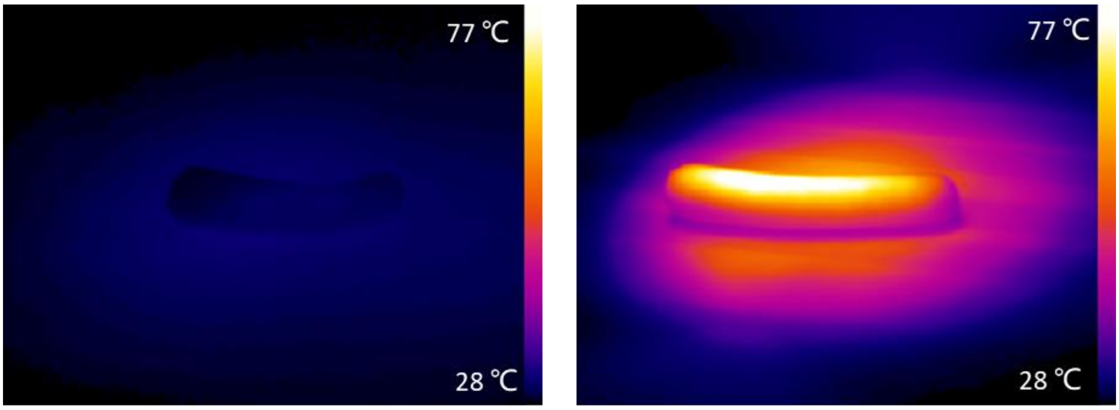

Supplementary Fig. 8. Infrared images of artificial muscle before and after light heating.

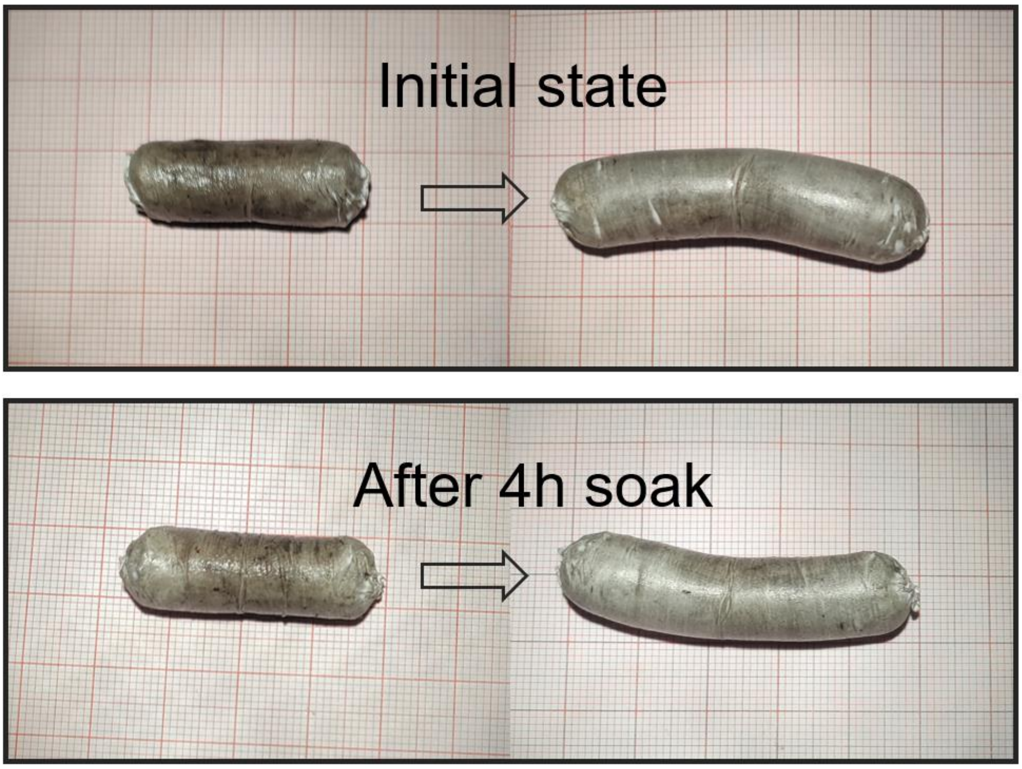

Supplementary Fig. 9. The artificial muscle can recover 80% of its initial elongation after 4h soak.

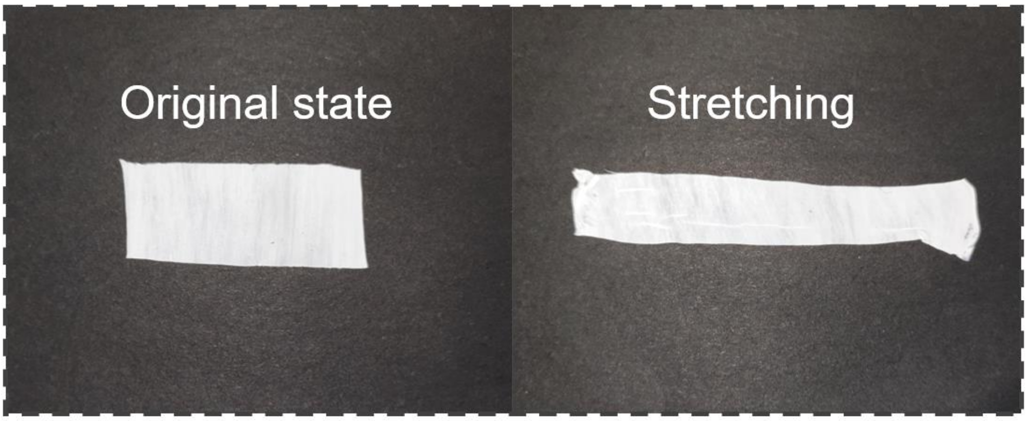

Supplementary Fig. 10. Optical image of PTFE film before and after stretching.
