# Supplementary material for: Miniaturized and untethered McKibben muscles based on photothermal-induced gas-liquid transformation
Source: Nat Commun. 2024 Feb 13;15:1329. doi: 10.1038/s41467-024-45540-4 (PMC10864313; doi:10.1038/s41467-024-45540-4)
Supplement: Supplementary file 3 — Description of Additional Supplementary Files [file 41467_2024_45540_MOESM3_ESM.pdf]

Description of Additional Supplementary Files

Supplementary Movie Legends

Supplementary Movie 1:Elongation of artificial muscle with temperature change

Supplementary Movie 2: Miniature artificial muscle walking device

Supplementary Movie 3: One step of the light driven earthworm-like artificial muscle

Supplementary Movie 4: Five steps of the light driven earthworm-like artificial muscle

Supplementary Movie 5: A flexible gripper made of artificial muscle

Supplementary Movie 6: A light controlled thruster
